# Paracrine Effects of IGF-1 Overexpression on the Functional Decline Due to Skeletal Muscle Disuse: Molecular and Functional Evaluation in Hindlimb Unloaded MLC/mIgf-1 Transgenic Mice

**DOI:** 10.1371/journal.pone.0065167

**Published:** 2013-06-03

**Authors:** Sabata Pierno, Giulia M. Camerino, Maria Cannone, Antonella Liantonio, Michela De Bellis, Claudio Digennaro, Gianluca Gramegna, Annamaria De Luca, Elena Germinario, Daniela Danieli-Betto, Romeo Betto, Gabriella Dobrowolny, Emanuele Rizzuto, Antonio Musarò, Jean-François Desaphy, Diana Conte Camerino

**Affiliations:** 1 Section of Pharmacology, Department of Pharmacy & Drug Sciences, University of Bari “Aldo Moro”, Bari, Italy; 2 Department of Biomedical Sciences, University of Padova, Padova, Italy; 3 Institute of Neuroscience, National Research Council, Padova, Italy; 4 Institute Pasteur Cenci-Bolognetti, DAHFMO-Unit of Histology and Medical Embryology, IIM, Istituto Italiano di Tecnologia, Sapienza University of Roma, Roma, Italy; 5 Department of Mechanical and Aerospace Engineering, IIM, Sapienza University of Roma, Roma, Italy; Cinvestav-IPN, Mexico

## Abstract

Slow-twitch muscles, devoted to postural maintenance, experience atrophy and weakness during muscle disuse due to bed-rest, aging or spaceflight. These conditions impair motion activities and can have survival implications. Human and animal studies demonstrate the anabolic role of IGF-1 on skeletal muscle suggesting its interest as a muscle disuse countermeasure. Thus, we tested the role of IGF-1 overexpression on skeletal muscle alteration due to hindlimb unloading (HU) by using MLC/mIgf-1 transgenic mice expressing IGF-1 under the transcriptional control of MLC promoter, selectively activated in skeletal muscle. HU produced atrophy in soleus muscle, in terms of muscle weight and fiber cross-sectional area (CSA) reduction, and up-regulation of atrophy gene MuRF1. In parallel, the disuse-induced slow-to-fast fiber transition was confirmed by an increase of the fast-type of the Myosin Heavy Chain (MHC), a decrease of PGC-1α expression and an increase of histone deacetylase-5 (HDAC5). Consistently, functional parameters such as the resting chloride conductance (gCl) together with ClC-1 chloride channel expression were increased and the contractile parameters were modified in soleus muscle of HU mice. Surprisingly, IGF-1 overexpression in HU mice was unable to counteract the loss of muscle weight and the decrease of fiber CSA. However, the expression of MuRF1 was recovered, suggesting early effects on muscle atrophy. Although the expression of PGC-1α and MHC were not improved in IGF-1-HU mice, the expression of HDAC5 was recovered. Importantly, the HU-induced increase of gCl was fully contrasted in IGF-1 transgenic mice, as well as the changes in contractile parameters. These results indicate that, even if local expression does not seem to attenuate HU-induced atrophy and slow-to-fast phenotype transition, it exerts early molecular effects on gene expression which can counteract the HU-induced modification of electrical and contractile properties. MuRF1 and HDAC5 can be attractive therapeutic targets for pharmacological countermeasures and then deserve further investigations.

## Introduction

The absence of gravitational loading, which occurs during physical inactivity due to prolonged bed rest, aging or spaceflight, predisposes humans to severe failure of vital tissues, such as cardiovascular system, bone and skeletal muscle. Skeletal muscle suffers from atrophy and functional decline, in terms of muscle weakness and increased fatigability, which lead to altered motor performance [1]. The anti-gravity slow-twitch muscles are the most affected ones, since in response to reduced neuromuscular activity, a remodeling of their biochemical and contractile properties toward a faster phenotype takes place [2]. Remodeling involves activation of intracellular signaling pathways and consequent genetic re-programming, which may also lead to the progressive muscle degradation [3]. Similar skeletal muscle alterations are observed in the hindlimb unloaded (HU) rodent model. This latter is a validated ground-based model to mimic and to study the effects of microgravity on skeletal muscle function and to evaluate effective countermeasures to ensure muscle performance [4]. In rat and mouse soleus (Sol) muscle, HU produces severe atrophy and a slow-to-fast phenotype transition as shown by the modification of myosin heavy chain (MHC) isoform distribution. Accordingly, due to the modified functional demands, the expression and function of proteins involved in the control of muscle excitability, excitation–contraction coupling, energy metabolism, and contractile properties are changed toward those of fast phenotype [5]–[9]. Indeed, compared to control Sol muscle, a lower cytosolic calcium level is found in the HU Sol muscle [7], [10]. Interestingly, an increase of activity and expression of the ClC-1 muscle chloride channel, the channel responsible for the resting chloride conductance (gCl) is observed in HU animals toward the value typical of fast muscles. As already shown, this is important for muscle function, since gCl is pivotal for sarcolemma stability and for the fine tuning of the electrical and contractile properties. The modification of gCl in HU Sol muscle precedes the changes in MHC isoforms, suggesting that it may also represent a key determinant of the phenotype transition [7].

In previous studies, we used the HU rodent to assess the potential efficacy of pharmacological treatments at the aim to find effective countermeasures to ameliorate muscle atrophy, the phenotype transition as well as the oxidative stress typical of muscle disuse. Thus, the antioxidant trolox did not prevent muscle atrophy but significantly attenuated the phenotype transition [7], while the amino-acid taurine had positive effects on the phenotype transition and mitigated Sol muscle atrophy molecular signaling [11]. In the present study, we explored the effects of insulin-like growth factor (IGF-1), which is involved in the regulation of muscle mass through the activation of specific signaling pathways [12]–[15]. Indeed, previous studies have demonstrated that localized muscle IGF-1 transgene expression in mice sustains hypertrophy and regeneration in senescent skeletal muscle [16]. Moreover IGF-1 overexpression or chronic treatment were able to ameliorate muscle damage in animal models of neuromuscular diseases, such as muscular dystrophy and amyotrophic lateral sclerosis [17]–[19]. In support of its therapeutic potential, higher levels of IGF-1 were found in the hind limb muscles and plasma of mdx mouse during the regeneration period [20]. Importantly, an increase of gCl was observed in parallel, thereby supporting the view that ClC-1 channels can be modulated by IGF-1 in vivo and that their function can be a sensitive index of the growth and differentiation processes in the fast-twitch muscle fibers [20]. Accordingly, we previously demonstrated that IGF-1 acutely applied *in vitro* to rat skeletal muscle can modify the resting gCl through an effect on the Protein Kinase C/phosphatase pathway involved in the control of ClC-1 channel [21], [22]. IGF-1 increases the resting gCl in Sol muscle through activation of phosphatase, whereas this effect is less pronounced in EDL muscle, in which the gCl is already elevated due to basal phosphatase activity [22]. In accord with the phenotype transition, the growth factor is also less effective when applied in vitro on HU-Sol muscle. Other studies have shown a reduction of muscle IGF-1 level during spaceflight-induced muscle inactivity [23] or rodent muscle unloading [24], [25]. Altogether, these data suggests that IGF-1 may represent a possible countermeasure against muscle impairment in disuse.

In the present study, we thus evaluated if IGF-1 supplementation may protect against the modifications induced by HU process in skeletal muscle. As a novel approach, we applied HU (14 days) to MLC/mIgf-1 transgenic mice that over-express a tissue-restricted transgene encoding for a locally acting isoform of IGF-1 in skeletal muscle (mIgf-1) [16]. The MLC/mIgf-1 transgene is driven by skeletal muscle specific elements from the rat myosin light chain (MLC) locus. The mIGF-1 isoform is normally synthesized in adult skeletal muscles, and predominates in fast-twitch muscles. Our hypothesis was that the local expression of mIGF-1 may increase in HU Sol muscle due to the enhanced number of fast fibers occurring during the phenotype transition and may hamper fiber atrophy in an autocrine or paracrine manner. Thus, we performed a multidisciplinary study on the slow- and fast-twitch muscles of IGF-1-HU mice by examining an ample battery of parameters known to be modified during the muscle disuse and markers of skeletal muscle phenotype and function. In parallel, the expression of selected genes related to atrophy, phenotype determination, and IGF-1 signaling were also evaluated. Our results indicate that although IGF-1 overexpression was not able to counteract atrophy and MHC composition, it can rescue functional parameters such as resting gCl, the rheobase voltage and the contractile properties and the expression of key genes such as MuRF1 and HDAC5. Thus, this study may represent a starting point for the development of effective countermeasures against the disuse-induced muscle impairment.

## Materials and Methods

### IGF-1 Transgenic Mice. Animal Care and Hindlimb Unloading

MLC/mIgf-1 transgenic mice overexpressing muscle IGF-1 gene have been obtained as previously described [16].

The experiments have been performed in accordance with the Italian Guidelines for the use of laboratory animals, which conforms with the European Union Directive for the protection of experimental animals (2011/63/EU), and received approval from the Italian Health Department. Accordingly during the experiments all efforts were made to minimize suffering. Six-months-old transgenic (initially weighting 40 g) and Wild-Type (Charles River Laboratories, Calco, Italy) FVB mice (initially weighting 30 g) were housed individually in appropriate cages in an environmentally controlled room. The animals were randomly assigned to control (ground) and hindlimb unloaded (HU) experimental groups as follows: (1) WT ground mice (WT-G, n = 10); (2) 14-days hindlimb-unloaded WT mice (WT-HU, n = 10); (3) MLC/mIgf-1 ground transgenic mice overexpressing muscle IGF-1 (IGF-G, n = 10); (4) 14 day hindlimb-unloaded MLC/mIgf-1 transgenic mice overexpressing muscle IGF-1 (IGF-HU, n = 10). All mice had water ad libitum and received 8 g a day of standard rodent chow (Charles River, 4RF21). The food remaining on the day after was weighted to calculate daily food consumption. To induce muscle unloading, the animals of HU group were suspended individually in special cages for 2 weeks as described [7]. A thin string was linked at one extremity to the tail by sticking plaster and at the other extremity to the top of the cage. The length of the string was adjusted to allow the animals moving freely on the forelimbs, while the body was inclined at 30–40° from the horizontal plane. The ground WT and ground IGF-1 mice were maintained free in single cages for 14 days. At the end of suspension, the mice were unfastened from the string and deeply anesthetized by intraperitoneal injection of urethane (1.2 g/kg body weight) to allow removing of soleus (Sol), gastrocnemius (Gas), Tibialis Anterior (TA), and Extensor Digitorum Longus (EDL) muscles. The same muscles were also removed from WT and IGF-1 ground mice in the same conditions. Muscles were used immediately for the functional experiments or frozen in liquid nitrogen and stored at −80°C for other studies. After surgery, animals were euthanized by an overdose of urethane.

### Cross Sectional Area (CSA) Analysis

Muscles were frozen in liquid nitrogen in a slightly stretched position. Serial cross sections (8-µm thick) were cut in a cryostat microtome set at −24±2°C (Slee Pearson, UK). To measure the cross-sectional area (CSA) of individual fibers, muscle cryostat section were stained for laminin, a major component of the basal lamina. Digital photographs were taken of each muscle section and the CSA was automatically measured as the internal laminin-unstained area by the ImageJ software (NIH, freeware imaging software) [26]. A minimum of 300 fibers per muscle were measured.

### Analysis of MHC Isoform Content

Fiber typing was determined by immunofluorescence using combinations of the following monoclonal antibodies: BA-D5 that recognizes type 1 MHC isoform; SC-71 for type 2A MHC isoform; BF-F3, for type 2B MyHC isoform [27]. To detect the primary antibodies the following secondary antibodies were used: DyLight405 labeled goat anti mouse IgG, Fcc 2b subclass specific (115-475-207), to specifically detect BA-D5, DyLight488 labeled goat anti mouse IgG, Fcc 1 subclass specific (115-485-205), for SC71, and DyLight549 labeled goat anti mouse IgM (115-505-075), used to specifically detect BF-F3. Secondary antibodies were purchased from Jackson Immunoresearch, anti-HA, 16B12, from Covance, USA, and anti-myc, 9E10, from Roche. Pictures were collected with an epifluorescence Leica DM5000 B equipped with a Leica DFC 300 FX camera. Single-color images were merged with Adobe Photoshop CS2 (Adobe Systems Inc.) to obtain a whole muscle reconstruction [26].

### Ion Channel Resting Conductances Measured by the 2-intracellular Microelectrodes Technique

Extensor Digitorum Longus (EDL) and Soleus (Sol) muscles were dissected from wild-type and IGF-1 transgenic animals, immediately placed in a muscle containing bath immersed in normal or chloride-free physiological solution maintained at 30°C and perfused with 95% O_2_/5% CO_2_ [28], [29]. The normal physiological solution contained (in mM): NaCl 148, KCl 4.5, CaCl_2_ 2.0, MgCl_2_ 1.0, NaHCO_3_ 12.0, NaH_2_PO_4_ 0.44, glucose 5.5, and pH 7.2. The chloride-free solution was prepared by equimolar substitution of methylsulfate salts for NaCl and KCl and nitrate salts for CaCl_2_ and MgCl_2_. The cable parameters of myofiber sarcolemma were determined from the electrotonic potentials elicited by square wave hyperpolarizing current pulse of 100-ms duration, using two intracellular microelectrodes in current-clamp mode, as previously described [28], [29]. From the values of input resistance, space constants and time constant and assuming a myoplasmic resistivity of 140 Ω·cm, the fiber diameter (dcalc), the membrane resistance (Rm) and the total membrane capacitance (Cm) were then calculated. The reciprocal of Rm, from each fiber in normal physiological solution was assumed to be the total membrane conductance (gm) and the same parameter measured in chloride-free solution was considered to be the potassium conductance (gK). The mean chloride conductance (gCl) was estimated as the mean gm minus the mean gK [28], [29].

### Measure of the Mechanical Threshold for Contraction

The mechanical threshold (MT) for contraction was determined using a two-microelectrode point voltage clamp method in the presence of 3 µM tetrodotoxin, as described previously [30], [31]. The holding potential was set to −90 mV. Depolarizing current pulses of various durations (5–500 ms) were applied at a frequency of 0.3 Hz, while the impaled fiber was continuously inspected with a stereomicroscope. The command voltage was increased until contraction was visible and the threshold membrane voltage was read at this time from a digital sample-and-hold voltmeter. The threshold membrane potential *V* (mV) for each fiber was averaged at each pulse duration *t* (ms) and then mean values from 7–16 fibers were plotted against duration, giving a “strength-duration” relationship. A fit estimate of the rheobase voltage (*R*) and of the rate constant (1/τ) to reach the R was obtained by nonlinear least-squares algorithm using the following equation: *V = *[*H – R* exp (*t/τ*)]/[1 – exp (*t*/τ)], where *H* is the holding potential (mV), *R* is the rheobase (mV), and τ is the time constant. In the fitting algorithm, each point was weighed by the reciprocal of the variance of that mean *V* and the best-fit estimates of the parameters *R* and 1/τ were made [30]. We used this procedure to be able to incorporate all of our determination points and their associated errors into our estimate of *R* under each condition.

### Fluorescence Measurements of Resting Intracellular Ca^2+^ Concentration

Fluorescence measurements were performed on small bundles of five to ten fibers lengthwise dissected from mice EDL and Sol muscles, as described elsewhere [10]. The muscle fibers were incubated with the fluorescent calcium probe fura-2 for 45–60 min at 22°C in physiological solution containing 5 µM of the acetoxymethyl ester (AM) form of the dye mixed to 10% (v/v) Pluronic F-127 (Molecular Probes, Leiden, The Netherlands). A QuantiCell 900 integrated imaging system (VisiTech International Ltd) was used to acquire pairs of background-subtracted images of the fura-2 fluorescence emission (510 nm) excited at 340 nm and 380 nm. The equation used to transform fluorescence ratio in [Ca^2+^]i values was [Ca^2+^]i = (R–Rmin)/(Rmax–R)·K_D_·β, where R is the ratio of fluorescence excited at 340 nm to that excited at 380 nm; K_D_ = 145 nM; β, Rmin and Rmax were determined in situ in ionomycin-permeabilized muscle fibers [10].

### Contraction Measurements

Briefly, isolated muscles (Sol or EDL muscle) were mounted in a temperature controlled chamber (30°C) filled with Krebs-Ringer bicarbonate buffer solution, continuously gassed with 95% O_2_/5% CO_2_ (pH = 7.3). One tendon is linked to a fixed clamp, while the other end is connected to the lever-arm of the isometric force transducer with a nylon thread. Electrical stimulation is provided with two platinum electrodes, which deliver constant current spikes of 300 mA [32]–[34]. Optimum length of the muscle was determined by twitch force from supramaximal stimulation. To evaluate time to peak (TTP) and 1/2 relaxation time (½RT) the muscle was stimulated with three single pulses of 500 ms. Tetanic force was measured with two stimuli at 180 Hz for EDL and 80 Hz for Sol using a 100-ms pulses delivered through the parallel platinum electrodes. Specific force was then obtained as maximum force divided by the Cross Sectional Area digitally calculated as previously described [33].

### Quantitative Real-time PCR Analysis

For each muscle sample, total RNA was isolated with RNeasy Fibrous Tissue Mini Kit (Quiagen) and quantified by using a spectrophotometer (ND-1000 NanoDrop, Thermo Scientific) [26]. Total RNA (400 ng) was used for reverse transcription. Synthesis of cDNA was performed by using random hexamers (annealed 10 min, 25°C) and Superscript II reverse transcriptase (Invitrogen–Life Technologies) incubated at 42°C for 50 min. Real-time PCR was performed in triplicate using the Applied Biosystems Real-Time PCR 7500 Fast system. Each reaction was carried on as singleplex reaction. The setup of reactions consisted 8 ng of cDNA, 0.5 µl of primer and probe set, 5 µl of TaqMan Fast Universal PCR master mix No AmpErase UNG (2x) (Applied Biosystems) and H_2_O nucleotide free for a final volume of 10 µl. Under the following PCR conditions: step 1, 95°C for 20 s; step 2, 95°C for 3 s; and step 3, 60°C for 30 s; steps 2 and 3 were repeated 45 times. The results were compared with a standard curve and normalized to expression of the housekeeping gene (HPRT1 TaqMan hydrolysis primer and probe gene expression assays were ordered by Applied Biosystems with the following assay IDs: Insulin receptor substrate Assay ID: Mm_01278327_m1; ryanodine receptor1 Assay ID: Mm_01175211_m1; calcium/calmodulin-dependent protein kinase II gamma Assay ID: Mm_00618054_m1; calcium channel, voltage-dependent, L type, alpha 1S subunit Assay ID: Mm_00489257_m1; protein phosphatase 3 Assay ID: Mm01317678_m1; ATPase, Ca^2+^ transporting, cardiac muscle, slow twitch 2 Assay ID: Mm_01201431_m1; ATPase, Ca^2+^ transporting, cardiac muscle, fast twitch 1 Assay ID: Mm_01275320_m1; ATPase, Na^+^/K^+^ transporting, alpha 2 polypeptide Assay ID: Mm_00617899_m1; ATPase, Na^+^/K^+^ transporting, alpha 1 polypeptide Assay ID: Mm_00523255_m1; myocyte enhancer factor 2d Assay ID: Mm_00504931_m1; myocyte enhancer factor 2c Mm_01340842_m1; histone deacetylase 5 Assay ID: Mm01246076_m1; peroxisome proliferative activated receptor, gamma, co-activator 1 alpha Assay ID: Mm_01208835_m1; muscle RING-finger protein-1 Assay ID: Mm_01185221_m1; Atrogin1 Assay ID: Mm_00499523_m1; hypoxanthine guanine phosphoribosyl transferase Assay ID:Mm00446968_m1. Some primer and probe sequences designed ATP-binding cassette, sub-family C (CFTR/MRP), member 9 probe: catagctcatcgggttc primer star:cggatcgcacggtcgta primer stop:taaccaggtctgcagtcagaatg. Potassium inwardly rectifying channel, subfamily J, member 11 probe:ctggctcctagtgacctg primer star: ctcatcatctaccacgtcatcga primer stop:gtttctaccacgccttccaaga. Chloride channel 1 probe: attggctgagacacttgt primer star:tcatgctcggtgtccgaaa primer stop: caggcggtgcttagcaaga.

### Statistical Analysis

To compare the four experimental groups of animals, statistical analysis was performed using two-way analysis of variance (ANOVA) followed by ad-hoc Bonferroni’s t test. Comparison of means between two experimental conditions was done using unpaired Student’s t-test. A P value minor to 0.05 was considered statistically significant.

## Results

### Effects of HU on Health and Behavior of WT and MLC/mIgf-1 Transgenic Mice

HU mice were examined daily over the entire HU period for behavior, aspect of hairs and eyes, food and water consumption. No sign of stress or mortality were evidenced. Daily food consumption over 14 days HU did not differ significantly between WT-G and WT-HU mice as well as between IGF-G and IGF-HU mice (not shown). No modification in body weight gain was observed after 14-days HU in WT animals. Although the IGF-G mice were significantly heavier with respect to WT mice, likely due to the muscle hypertrophy, they showed a significant weight loss after 14-days of HU (Table 1).

**Table 1:** Body weight of wild-type and IGF-1 mice before and after 14-days HU.

	N mice	Initial weight (g)	Final weight (g)
WT	6	28.9±1.9	30.3±2.2
WT-HU	6	30.8±0.2	29.4±0.9
IGF-1	5	41.0±2.6a,b	39.8±2.0a,b
IGF-1-HU	7	39.3±3.0a,b	32.5±1.8*

2-way ANOVA/Bonferroni, Interaction F = 1.42, Genotype F = 12.51 (P<0.001), Time F = 1.87.

a vs. WT;

b vs. WT-HU (vP<0.01 or less);

* vs. respective initial weight (P<0.05).

### Effect of IGF-1 Local Overexpression on HU-induced Muscle Atrophy in MLC/mIgf-1 Transgenic Mice

Muscle atrophy is a major consequence of HU together with the slow-to-fast phenotype transition. We evaluated muscle atrophy in Sol, Gas, TA and EDL muscles by measuring the muscle weight as well as the fiber diameter and the fiber cross sectional area (CSA), as follows.

#### Muscle weight

**Figure 1:**
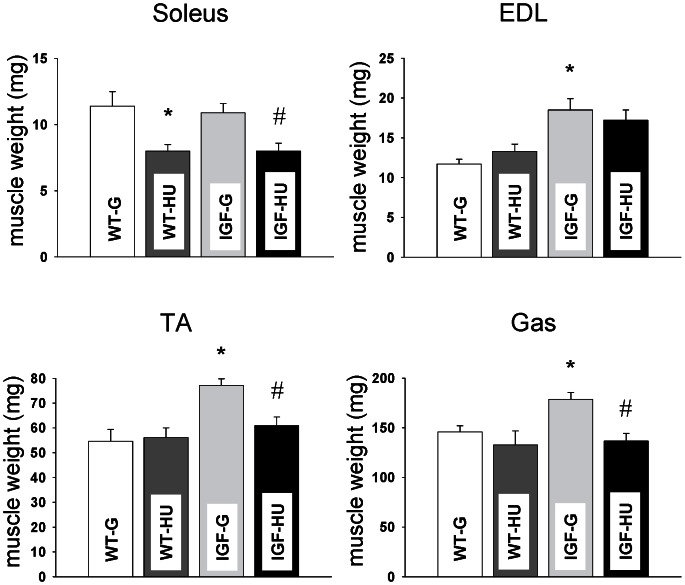
Effects of 14-days hindlimb unloading (HU) on muscle weight of Wild-Type and MLC/migf-1 transgenic mice. Each bar represents the mean ±S.E.M. of the muscle weight (in mg) of Soleus, extensor digitorum longus (EDL), tibialis anterior (TA) and gastrocnemious (Gas) muscles measured in 3–6 Wild-Type-ground (WT-G), Wild-Type-HU (WT-HU) and MLC/migf-1 transgenic mice before (IGF-G) and after HU (IGF-HU). Statistical analysis has been performed for each muscle type using Analysis of Variance (ANOVA) followed by Bonferroni’s t-test. Significantly different *with respect to WT-G and #with respect to IGF-G (at least P<0.05).

In WT mice, the Sol muscle showed a significant atrophy after 14 days of HU, as evidenced by the reduction of muscle weight (Fig. 1). A not significant reduction in Gas muscle weight was observed after HU, while TA and EDL muscle weights were not modified. Hypertrophy of the fast-twitch EDL, TA and Gas muscles was observed in IGF-1 overexpressing animals with respect to WT-G animals, whereas Sol muscle weight of IGF-G mice was similar to that of WT-G (Fig. 1). In IGF-1 mice, HU significantly decreased the weight of Sol, Gas and TA muscles.

#### Muscle fiber diameter

The fiber diameter of Sol and EDL muscles was calculated from cable parameters, as detailed in the Methods section. It was changed in accord to the muscle weight. In fact, fiber diameter in Sol muscle of WT-HU mice was decreased by 18% with respect to that of WT-G mice. No amelioration of fiber diameter was found in the Sol muscle of IGF-HU mice with respect to IGF-G, being significantly reduced from 44.6±1.7 µm (N, number of mice/n, number of fibers = 3/28) to 32±0.7 µm (N/n = 2/19) (P<0.005). In EDL muscles, no significant difference was found in fiber diameter between WT-G and WT-HU. However, fiber diameter was higher in EDL of IGF-G mice with respect to WT-G (59.6±2.8 µm, N/n = 3/24 vs. 50.2±2.3 µm, N/n = 3/25; P<0.005) and was significantly reduced after HU (44.4±1.5 µm, N/n = 3/37; P<0.001).

#### Muscle fiber Cross Sectional Area (CSA)

**Figure 2:**
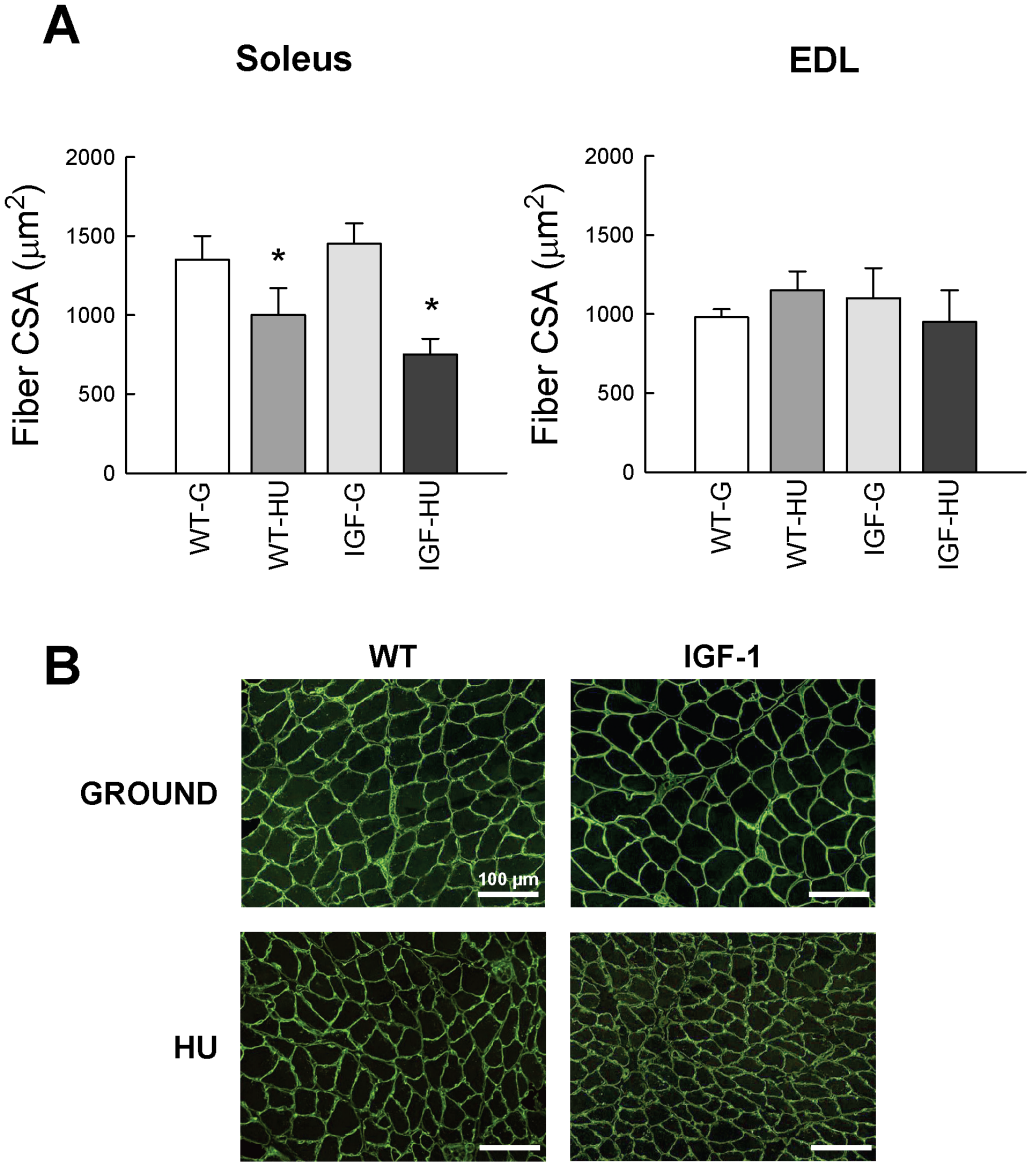
Cross sectional area (CSA) in skeletal muscle of Wild-Type and MLC/migf-1 transgenic mice during HU. **A.** CSA values measured in Soleus and EDL muscle fibers of ground-based WT (WT-G), WT-hindlimb unloaded (WT-HU), ground-based transgenic IGF-1 (IGF-G) and IGF-1 transgenic-HU (IGF-HU) mice. Bars represent the mean ± S.E.M. of 3–6 animals. Statistical differences were evaluated by Analysis of Variance (ANOVA) followed by Bonferroni’s t-test. Significantly different *with respect to WT-G and IGF-G (at least P<0.05). **B.** Immunofluorescence analysis of laminin localization in Soleus and EDL muscle fibers from the four groups of animals. The area inside the laminin staining was utilized to measure muscle fiber CSA.

Analysis of individual muscle fibers of Sol and of EDL muscles collected from WT-G, WT-HU, IGF-G and IGF-HU mice showed a significant 25% reduction of CSA in Sol muscle of WT-HU mice (Fig. 2). In accord with the lack of modification of Sol muscle weight in the IGF-G overexpressing mice with respect to WT-G, the CSA was not modified (Fig. 2). After HU, the CSA of Sol muscle fibers was greatly reduced by 50% in transgenic mice, suggesting that the paracrine activity of IGF-1 is not able to counteract the Sol muscle atrophy. The CSA of EDL muscle fibers was not modified after HU (Fig. 2).

### Myosin Heavy Chain Expression and Muscle Fiber Type Composition in WT and MLC/mIgf-1 Transgenic Mice during HU

**Figure 3:**
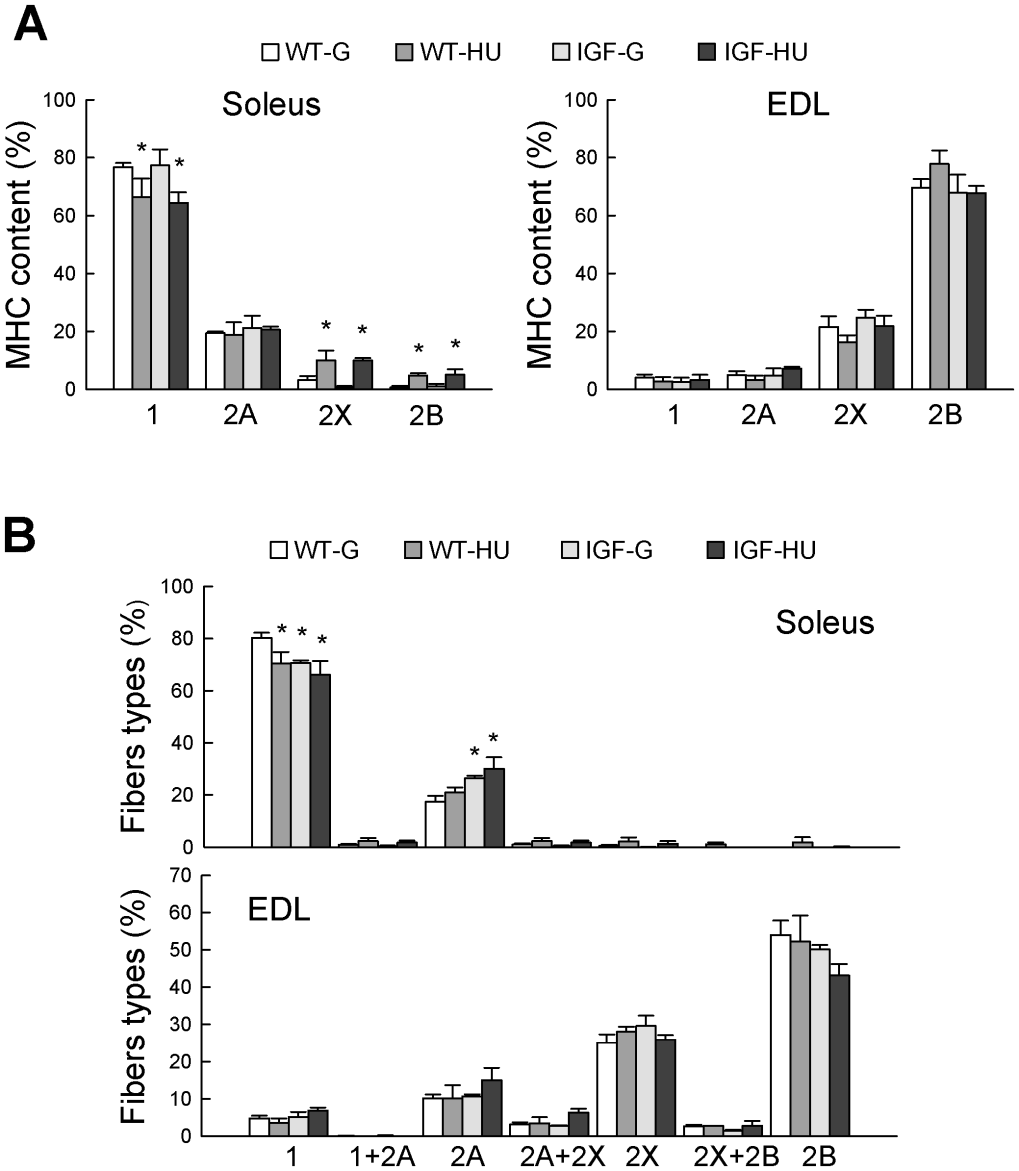
Myosin Heavy Chain (MHC) isoform composition in skeletal muscle of Wild-Type and MLC/migf-1 transgenic mice during HU. **A.** MHC values measured in Soleus and extensor digitorum longus (EDL) muscles from WT-ground (WT-G), WT-hindlimb unloaded (WT-HU), ground-based transgenic IGF-1 (IGF-G) and IGF-1 transgenic-HU (IGF-HU) mice. The MHC isoforms proportion was determined by densitometric analysis of SDS-PAGE gels. **B.** Fiber type composition determined by muscle cryosections. Bars represent the mean ± S.E.M. of 3–6 animals. Statistical differences were evaluated by Analysis of Variance (ANOVA) followed by Bonferroni’s t-test. Significantly different *with respect to WT-G (at least P<0.05).

We have previously demonstrated [6] that the muscle disuse causes a slow-to-fast phenotype transition, particularly in antigravity muscles, such as the Sol muscle. We measured the expression profile of myosin heavy chain (MHC) isoforms using SDS-PAGE of solubilized muscle fragments (Fig. 3A). The Sol muscle of WT animals contained 76.7±1.4% of slow type 1 MHC, 19.4±0.6% of 2A MHC, and a low amount of 2X and 2B MHC. After HU, Sol muscle showed a significant reduction of type 1 MHC protein and an increase of type 2X and 2B MHC isoforms, demonstrating the typical change of phenotype due to HU (Fig. 3A). No difference was found in Sol muscle of IGF-G with respect to WT-G mice. Overexpression of IGF-1 did not prevent the HU-induced changes of MHC composition. MHC isoform expression pattern was not significantly altered in the EDL muscle of both WT and transgenic HU animals.

To determine the fiber type compositions of Sol and EDL muscles, serial muscle cryosections were stained with monoclonal antibodies specific for type 1, 2A and 2B MHC subtypes [26]. Type 2X fibers were identified by the absence of reactivity with the three antibodies. Quantitative analysis validates the change of phenotype in HU-Sol muscle, with a significant reduction of type 1 fibers and a slightly increased proportion of pure 2A and 2X fibers (Fig. 3B). Also pure type 2B fibers appeared after HU. Although the proportion of mixed fibers is very small, fibers co-expressing type 1–2A, 2A-2X, and 2X-2B MHC isoforms were found in HU Sol muscle (Fig. 3B). Of note, over-expression of IGF-1, per se, determined an increase in type 2A fibers with a reduction in type 1 fibers in Sol muscle. No protection of the slow phenotype was observed in IGF-HU animals since type 1 fibers were still significantly lower and type 2A fibers were still significantly higher with respect to WT-G mice. The slight increase of type 2B fibers induced by HU was not found in IGF-1 overexpressing mice (Fig. 3B). No significant changes were found in the EDL muscle (Fig. 3B).

### Effects of IGF-1 Local Overexpression on the Resting Chloride Conductance in Muscle Fibers of MLC/mIgf-1 Transgenic Mice during HU

**Figure 4:**
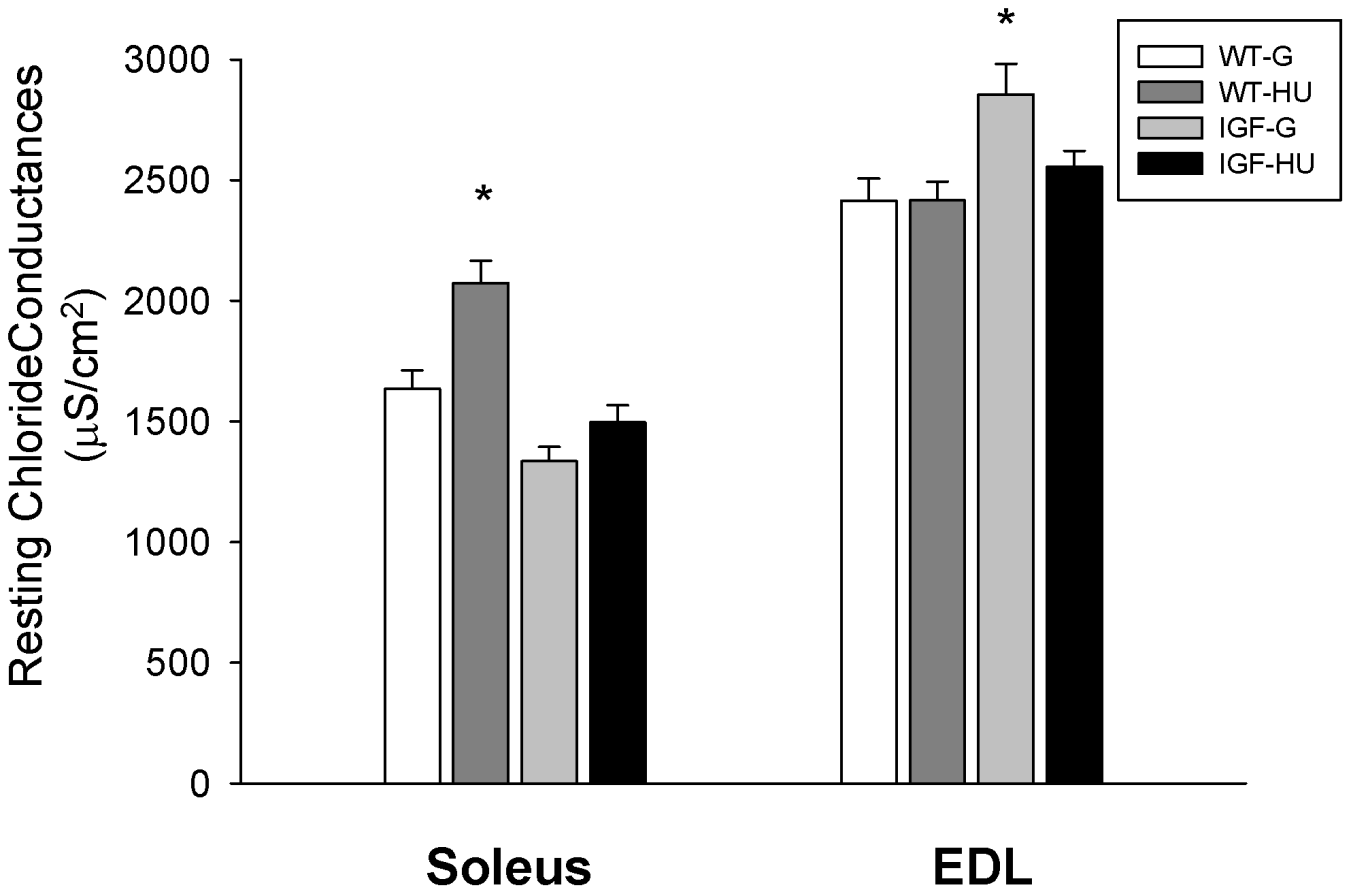
Resting chloride conductance measured in skeletal muscle of Wild-Type and MLC/migf-1 transgenic mice during HU. Each bar represents the mean value ± S.E.M. of the resting chloride conductance (gCl) measured in Soleus and Extensor Digitorum Longus (EDL) muscle fibers of ground-based WT (WT-G), WT-hindlimb unloaded (WT-HU), ground-based transgenic IGF-1 (IGF-G) and IGF-1 transgenic-HU (IGF-HU) mice. Bars represent the mean ± S.E.M. of 3–6 animals. Statistical analysis was performed using ANOVA followed by Bonferroni’s t-test. Significantly different *with respect to WT-G (at least P<0.05).

We have previously observed that the resting chloride conductance (gCl) is about 1.8 times higher in the EDL muscle fibers with respect to the Sol muscle fibers of mice [7]. After 14 days of HU the gCl value increased by 27% in Sol muscle of WT mice in line with the transition toward a faster phenotype. In Sol muscle of IGF-G mice the resting gCl was only slightly lower with respect to WT-G. Interestingly, the resting gCl in Sol muscle was restored in the IGF-HU mice, being similar to that of WT-G (Fig. 4). Resting gCl was unchanged in the EDL muscle of WT animals after HU, but was significantly higher in the EDL of IGF-G mice with respect to WT-G. In spite of this, the resting gCl found in IGF-HU mice was similar to that of WT-G (Fig. 4).

### Effects of IGF-1 Local Overexpression on the Mechanical Threshold of Skeletal Muscle of MLC/mIgf-1 Transgenic Mice during HU

**Figure 5:**
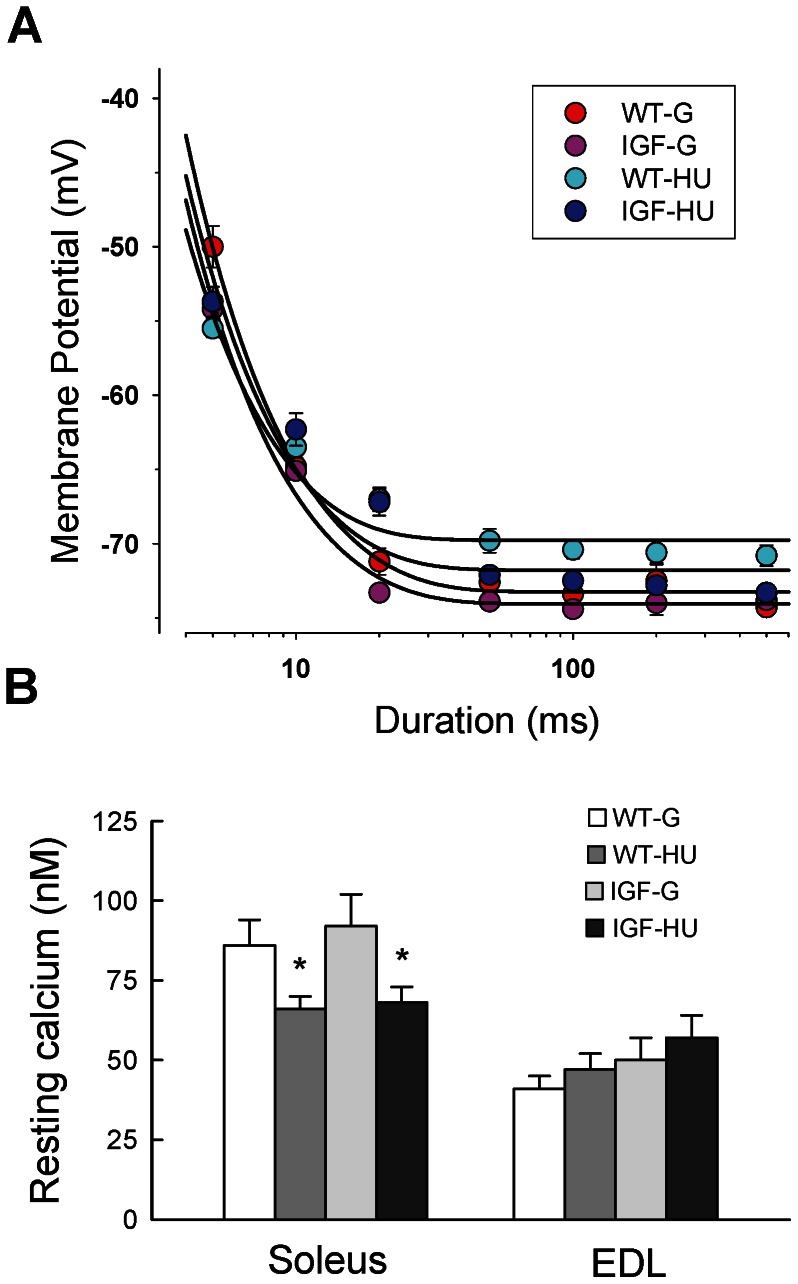
Mechanical threshold for contraction and resting cytosolic calcium level in skeletal muscle of Wild-Type and MLC/migf-1 transgenic mice during HU. **A.** Mechanical threshold (MT) for contraction measured in Soleus (Sol) muscle fibers of WT-ground (WT-G), WT-hindlimb unloaded (WT-HU), ground-based transgenic IGF-1 (IGF-G) and IGF-1 transgenic-HU (IGF-HU) mice. Each point, expressed as mean value ± S.E.M. from 9–20 fibers, show the voltage potential for fiber contraction at each pulse duration measured in different experimental conditions. The values have been fitted to the equation shown in the Methods section to obtain the strength–duration curves and the calculated values of rheobase (R) are given in the Results section. **B.** Each bar represents the mean ± S.E.M. of resting intracellular calcium level (in nM) from 9 to 40 fibers of 3–6 mice of the four groups. Statistical differences were evaluated by Analysis of Variance (ANOVA) followed by Bonferroni’s t-test. Significantly different *with respect to WT-G and IGF-G (at least P<0.05).

The Mechanical Threshold (MT) for contraction, that is the smallest voltage able to elicit contraction, is an integrative measure of the excitation-contraction coupling mechanism. The MT was determined at different current pulse durations in order to obtain a threshold-duration relationship, the fit of which allows the calculation of the rheobase (R) and of the time constant (τ) to reach R. As already observed, Sol muscle fibers need significantly less depolarization to contract with respect to the fast-twitch EDL muscle fibers, the R values being –73.3±0.3 mV and -69.0±0.3 mV, respectively (P<0.001). After 14 days of HU, the MT in Sol muscle was significantly shifted toward less negative voltages after HU (–69.7±0.6 mV), in line with the shift toward a faster phenotype (Fig. 5A). IGF-1 overexpression did not modify the R value in Sol muscle in control condition, but partially prevented the modification of MT Sol muscle induced by HU, its value (–71.8±1.0 mV) being not significantly different with respect to that of Sol muscle of WT-G (Fig. 5A). No modification was found in the R value of EDL muscle in all the experimental conditions (data not shown).

### Effects of IGF-1 Local Overexpression on the Resting Cytosolic Calcium Concentration in Skeletal Muscle of MLC/mIgf-1 Transgenic Mice during HU

As previously described in rats and mice [7], [10], the cytosolic calcium concentration at rest (restCa) depends on the muscle phenotype, being about two-fold higher in the slow-twitch Sol muscle compared to the fast-twitch EDL muscle (Fig. 5B). HU caused a decrease of restCa in WT Sol muscle as expected from the shift toward a faster phenotype. The overexpression of IGF-1 did not affect restCa of Sol and EDL muscles in ground conditions and did not prevent the HU-induced decrease of restCa in Sol muscle (Fig. 5B).

### Effects of mIGF-1 Local Overexpression on Skeletal Muscle Contractile Parameters Measured in MLC/mIgf-1 Transgenic HU Mice

**Figure 6:**
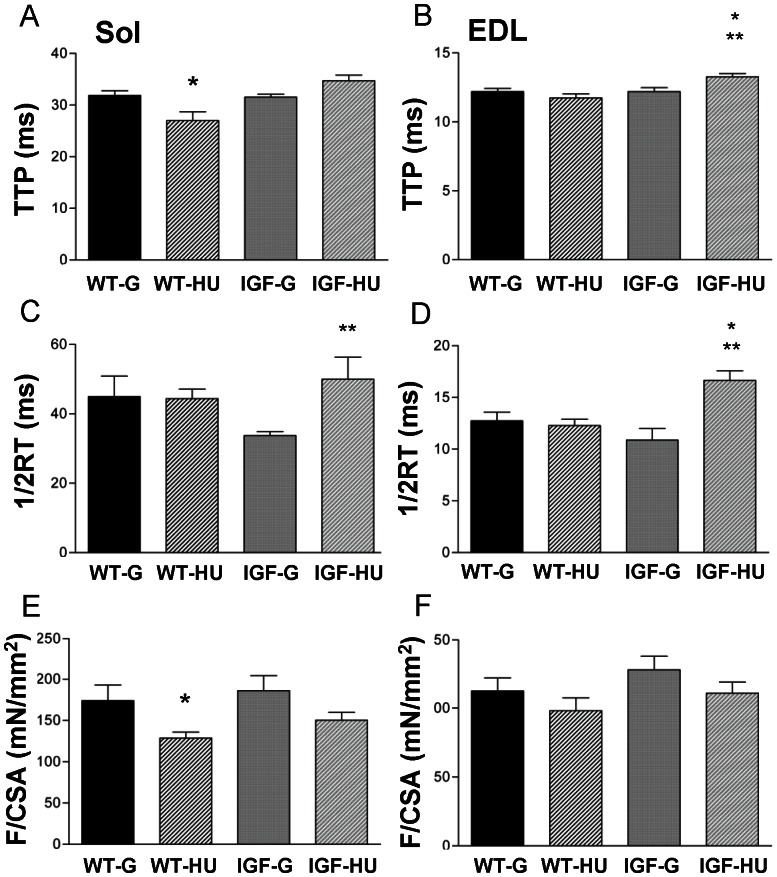
Contractile parameters measured in skeletal muscle of Wild-Type and MLC/migf-1 transgenic mice during HU. Time to peak (**A, B**), half relaxation time (**C, D**) and maximum specific force (**E, F**) in Soleus (Sol) and Extensor Digitorum Longus (EDL) muscle of ground-based WT (WT-G), WT-hindlimb unloaded (WT-HU), ground-based transgenic IGF-1 (IGF-G) and IGF-1 transgenic-HU (IGF-HU) mice. Values represent the mean ± SEM from 6 animals. Statistical analysis was performed using ANOVA followed by Bonferroni’s t-test. Significantly different *with respect to WT-G (at least P<0.05). Significantly different **with respect to IGF-G (at least P<0.05).

We evaluated the effects of HU and IGF-1 transgene on muscle contractile parameters, including twitch time to peak (TTP) and relaxation time (½RT), as well as the specific force (F/CSA). In Sol muscle of WT mice exposed to 14 days HU, TTP was significantly shorter compared to ground animals (Fig. 6A). The HU-induced shortening of TTP was prevented by in IGF-1 overexpression in transgenic mice (Fig. 6A). The ½RT was unchanged in Sol muscle of WT mice after HU. However an increase of this parameter was found in Sol muscles of IGF-HU mice (Fig. 6C). Notably, the specific force mean value was lower in Sol muscle of WT-HU mice with respect to WT-G, suggesting a decrease of specific force in these mice (Fig. 6E). This decrease was slightly prevented in Sol of IGF-HU mice. No effect of HU was observed in the EDL muscle of WT animals, while a significant increase of TTP and ½RT was found in IGF-HU mice (Fig. 6B,D,F).

### Gene Expression Analysis in Skeletal Muscle of MLC/mIgf-1 Transgenic HU Mice

**Figure 7:**
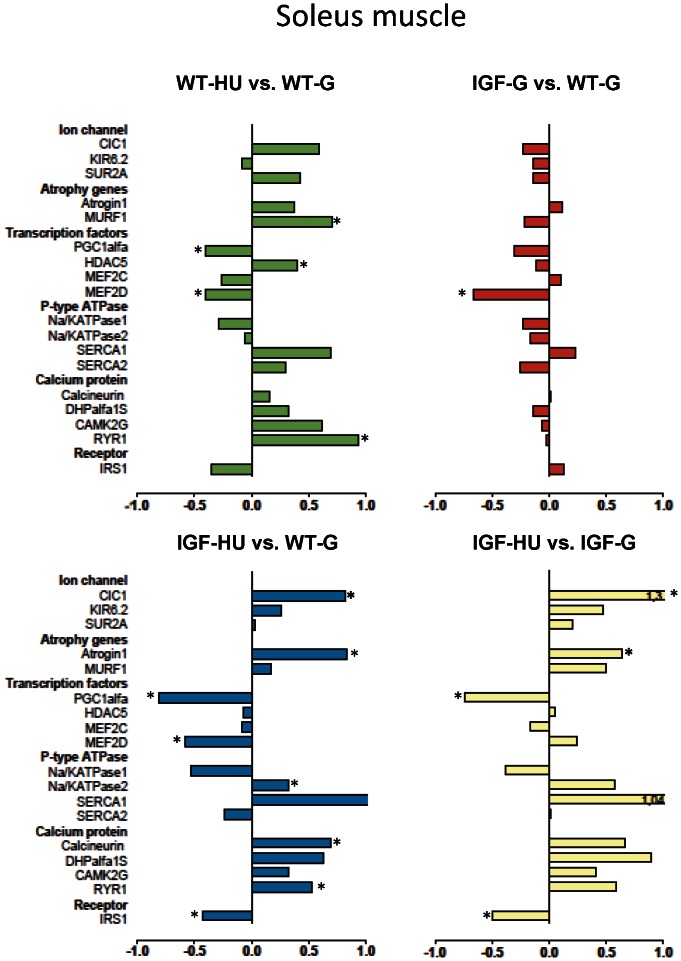
Gene expression modification in Soleus muscle induced by HU and effects of IGF-1 overexpression. Transcript levels were determined by real-time PCR for selected genes, classified on the basis of the functional role of the protein they encode. The numbers on the abscissa indicate the fold change in gene expression normalized for housekeeping gene. The bars in green show the variation between WT-hindlimb unloaded (WT-HU) vs. WT-ground (WT-G) mice, the bars in red show the variation between ground-based IGF-1 transgenic (IGF-G) vs. WT-ground (WT-G) mice, the bars in blue compare the variation between IGF-1 transgenic HU (IGF-HU) vs. WT-ground (WT-G) mice and the bars in yellow the variation between IGF-1 transgenic HU (IGF-HU) vs. ground-based IGF-1 transgenic (IGF-G) mice. Statistical differences were evaluated by Analysis of Variance (ANOVA) followed by Bonferroni’s t-test. (*at least P<0.05).

**Figure 8:**
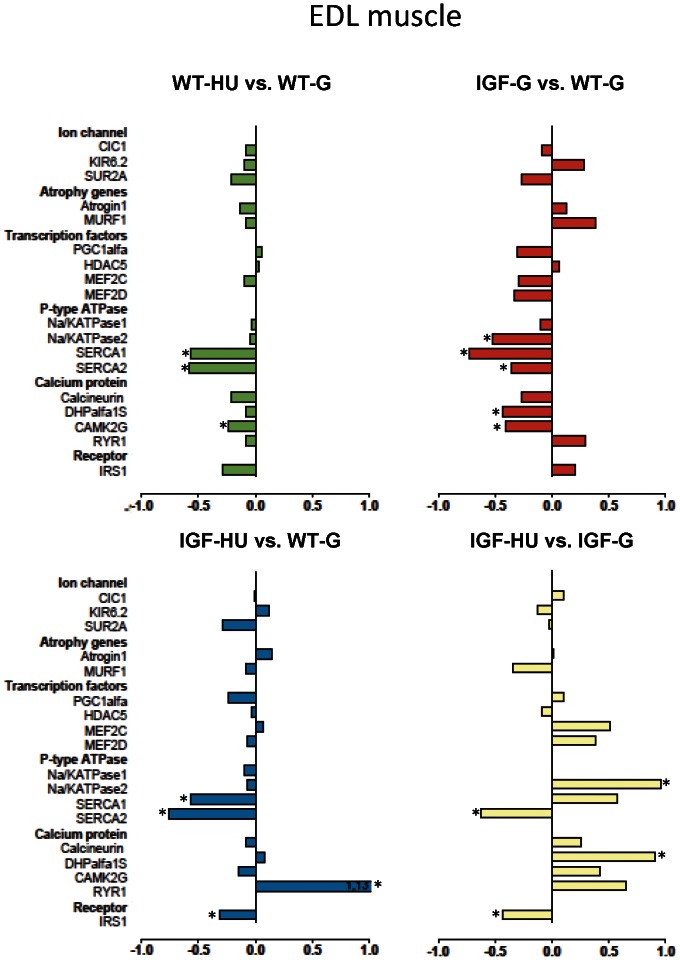
Gene expression modification in EDL muscle induced by HU and effects of IGF-1 overexpression. Transcript levels were determined by real-time PCR for selected genes, classified on the basis of the functional role of the protein they encode. The numbers on the abscissa indicate the fold change in gene expression normalized for housekeeping gene. The bars in green show the variation between WT-hindlimb unloaded (WT-HU) vs. WT-ground (WT-G) mice, the bars in red show the variation between ground-based IGF-1 transgenic (IGF-G) vs. WT-ground (WT-G) mice, the bars in blue compare the variation between IGF-1 transgenic HU (IGF-HU) vs. WT-ground (WT-G) mice and the bars in yellow the variation between IGF-1 transgenic HU (IGF-HU) vs. ground-based IGF-1 transgenic (IGF-G) mice. Statistical differences were evaluated by Analysis of Variance (ANOVA) followed by Bonferroni’s t-test. (*at least P<0.05).

To determine the adaptation changes of gene expression due to microgravity exposure and to evaluate the effects of IGF-1, we used Real-time PCR analysis. By this analysis we quantified the shift in mRNA levels of selected group of genes involved in muscle atrophy and plasticity. The results show the effects of HU in WT and in IGF-1 transgenic mice and the effects of IGF-1 overexpression in Sol (Fig. 7) and EDL (Fig. 8) muscles, as described hereafter.

#### Atrophy related genes expression

As expected, MuRF1 and atrogin-1 transcripts, associated with the ubiquitin-proteasome system, and specifically activated in muscle atrophy, were increased in Sol muscle after HU (Fig. 7). While the expression of these genes was not affected by IGF-1 overexpression in ground animals, atrogin-1 was still elevated but MuRF1 mRNA expression was partially recovered in IGF-HU mice. The expression of these genes was not modified in the EDL muscle after HU (Fig. 8).

#### Ion channels expression

The expression of different muscle ion channels was evaluated here. No difference was observed between WT-G and IGF-G mice in Sol and EDL muscles (Fig. 7 and Fig. 8). As already demonstrated in rats and mice [6], [7], in line with the slow-to-fast phenotype transition, the expression of ClC-1 chloride channel, which is the main contributor to gCl, was increased in Sol muscle of WT mice during HU. The expression of ClC-1 channel was also significantly higher in Sol muscle of transgenic mice after HU, suggesting no beneficial effect of IGF-1. This indicates that the prevention of HU-induced gCl increase in transgenic mice may be due to an IGF-1-dependent post-transcriptional regulation. No significant differences were found in the expression of Kir6.2 and SUR2A genes, encoding two KATP channel subunits, after HU either in Sol or in EDL muscles of WT or IGF-1 transgenic mice (Fig. 7 and Fig. 8).

#### Transcription factors and muscle phenotype regulators expression

Peroxisome proliferator-activated receptor gamma coactivator-1 alpha (PGC-1α) has emerged as a key regulator of mammalian energy metabolism and fiber type-switching in skeletal muscle. A reduced expression of PGC-1α was found in Sol muscles of WT mice after HU, likely related to the slow-to-fast phenotype transition (Fig. 7). Overexpression of IGF-1 was not able to prevent PGC-1α down-regulation induced by HU in Sol muscle of IGF-1 transgenic mice. No major changes were observed in EDL muscle (Fig. 8). Myocyte enhancer factor 2c (MEF2c), an important modulator of muscle gene expression, was not modified in any experimental conditions in both muscle types. Instead, MEF2d was significantly reduced in Sol muscle of WT mice after HU as well as in IGF-G and IGF-HU mice. This factor was not modified in the EDL muscle. The expression level of the histone deacetylase 5 (HDAC5), involved in the regulation of fast gene expression, was not affected by IGF-1 overexpression in both WT Sol and WT EDL. Expression of HDAC5 was significantly increased in Sol muscle of WT-HU mice but fully restored in IGF-HU mice. HDAC5 was unchanged in EDL muscle.

#### Calcium-dependent proteins, P-type ATPase and Insulin Receptor Substrate-1 expression

HU induced a not significant increase of sarcoplasmic reticulum Ca^2+^-ATPase (SERCA1, the fast SERCA isoform) expression in Sol of WT animals, in agreement with the slow-to-fast transition (Fig. 7). This effect was still evident in Sol muscle of IGF-HU mice. In EDL of WT mice, HU caused a significant reduction of both SERCA1 and SERCA2 isoforms, which is the most relevant effect produced by HU in EDL (Fig. 8). In contrast, in EDL muscle of IGF-1 transgenic mice, HU caused a down-regulation of SERCA2 only, whereas it induced up-regulation of SERCA1. HU was without major effects on the expression of the α1S dihydropyridine receptor (DHPR) isoform in Sol and EDL of WT mice, whereas its expression was significantly lower in the EDL muscle of IGF-G compared to WT-G. The transcript levels of ryanodine receptor 1 (RyR1) were similar in IGF-G and WT-G mice. HU induced a significant increase of RyR1 transcript both in WT and in IGF-1 transgenic Sol, while an increase was evident in IGF-1 transgenic EDL but not in WT-EDL. The Na/K ATPase1, important in the control of resting potential, was not significantly modified in Sol muscle but was significantly decreased in EDL muscle of IGF-G mice. Calcineurin, a protein phosphatase involved in the maintenance of the slow muscle fiber gene program and hypertrophy, was not modified in Sol muscle after HU or by IGF-1 overexpression, but was significantly increased in Sol muscle of IGF-HU mice. The expression of this phosphatase was not modified in the EDL muscle. The gamma subunit of calcium/calmodulin-dependent protein kinase type II (CaMKII) is involved in the regulation of SR Ca^2+^ transport in slow-twitch muscles. Its expression was unaltered in Sol muscle, but was decreased in EDL muscle of both WT-HU and IGF-G mice. Insulin receptor substrate-1 (IRS-1), which transmit the signals from IGF-1 receptor to downstream proteins, was slightly decreased after HU in Sol and EDL of WT animals and significantly decreased in both muscles of IGF-HU mice.

## Discussion

The results from this study add new insights into the mechanisms underlying the modifications in slow-twitch skeletal muscle due to inactivity and clarify the potential paracrine effect of IGF-1 overexpression in modulating these alterations. A few studies suggested that the mechanism of muscle wasting caused by disuse may involve a decreased IGF-1/PI3K signaling, with an increase of the expression of MuRF1 and atrogin-1 via modulation of the AKT/forkhead box O1 (FOXO) cascade [35], [36]. During severe disuse-induced-atrophy due to spaceflight or HU, the IGF-1 pathway alteration is accompanied by elevated pro-inflammatory signaling, including the NFκB pathway, which likely contributes to muscle impairment [37]. In this situation, stimulation of the IGF-1 signaling pathway may have a beneficial role in the regulation of the balance between hypertrophy and atrophy [38]. In fact, various studies have demonstrated that exogenous IGF-1 has the ability to induce muscle hypertrophy and may have therapeutic value in treating muscle degenerative diseases, sarcopenia, disuse-mediated skeletal muscle atrophy and bone osteopenia [39]–[43], although the actual effectiveness is still controversial [44], [45]. The rationale of our study includes the known biological effects of IGF-1 on chloride channel conductance (gCl), calcium homeostasis and mechanical threshold for contraction. In previous studies, IGF-1 was shown to positively modulate these targets of muscle disuse through the regulation of protein synthesis or post-transcriptional mechanisms [17], [20], [46].

In the present study, we observed that mIGF-1 overexpression has limited effects in the prevention of HU-induced muscle atrophy, since Sol muscle weight and CSA were lower in the IGF-HU mice compared to loaded mice. It also appeared that TA and Gas muscles that were less sensitive to HU in WT mice, showed a significant weight reduction in IGF-HU mice. The significant reduction of IGF-1 transgenic mouse body weight after HU likely resulted from the generalized reduction of muscle mass. This unexpected finding suggests that, after HU, either slow or fast muscles are no more sensitive to the hypertrophic action of IGF-1. Such condition may be related to the reduction of IRS-1 activity in slow and fast muscles after HU, due to both reduced IRS-1 transcript levels, as we observed here, and increased IRS-1 protein degradation as suggested by others [36]. However, the real-time PCR analysis showed that IGF-1 overexpression was able to oppose the HU-induced increase of MuRF1 transcript level in mouse Sol muscle. Interestingly, we previously showed that the increased expression of MuRF1 in HU rats was also mitigated by the exogenous administration of the amino acid taurine [11]. Therefore, MuRF1 appears to be a very sensitive target of potential countermeasures. MuRF1 is a RING-finger-dependent ubiquitin ligase that regulates proteasomal degradation of troponin and of other sarcomeric-associated proteins involved in mechanical loading and stretch, as well as myosin heavy chains [47]–[49]. Thus, it is directly involved in various aspects of contractile function regulation. Accordingly, the maintenance of low levels of MuRF1 in Sol muscle of IGF-HU mice may play a role in the observed amelioration of contractile properties, likely as a consequence of a reduced degradation of contractile proteins. In contrast to MuRF1, the HU-induced increased expression of atrogin-1 was not mitigated by IGF-1 overexpression. It is however well established that the two ubiquitin-ligases can function independently of each other, via activation of specific signaling pathways such as that of TNF and NFκB, and may target different proteins [50], [51]. Thus, our results suggest that IGF-1 may balance the degradation of proteins involved in contraction through its specific action on MuRF1, without impeding a generalized muscle atrophy. Future studies are warranted to verify this hypothesis.

We also observed that IGF-1 exerted a specific action on the expression of genes involved in the determination of slow-twitch phenotype, suggesting that the growth factor may promote a slow-phenotype genes program, which is however insufficient to fully protect the muscle from the transition. For instance, the expression of calcineurin, a calcium-dependent serine-threonine phosphatase involved in maintaining slow and oxidative properties in adult slow-twitch muscle fibers, was increased in Sol muscle of IGF-1 HU animals. In parallel, IGF-1 did not restore the expression of the PGC-1α, claimed to be a major downstream and final modulator of slow genes programs [52]. The comprehension of the pathways that contrast the IGF-1-activated calcineurin signaling may help for better understand the reason of the lack of effect on the HU-induced phenotype transition.

In agreement with the potential initial effect of IGF-1 in the phenotype control, we found that the HU-induced increase in gCl in Sol muscle is prevented by IGF-1 overexpression, suggesting a role of IGF-1 in the modulation of the early events triggering the transition. The lack of effect of IGF-1 on ClC-1 channel gene expression indicates a post-transcriptional action of the growth factor through a mechanism still to be clarified, but which may likely involve the mechanism of channel modulation via PKC, which we previously found to be modified during HU. Since MuRF1 has been shown to inhibits the activity of the calcium-independent PKCε in cardiomyocytes [53], we speculate that elevated MuRF1 during HU may lead to PKCε inhibition, which contributes to the increase of gCl and that IGF-1, by maintaining low MuRF1 level, can allow a PKC activity sufficient to maintain a low gCl in Sol muscle. Again, these hypotheses point out MuRF1 as an attractive therapeutic target for disuse-associated skeletal muscle impairment [48].

Interestingly, in Sol muscle of IGF-HU mice, we found a full restoration of the expression of histone deacetylase 5 (HDAC5), a protein implicated in muscle plasticity [54]. HDAC5 protein level is higher in fast muscles compared to slow [55], in this latter the low HDAC5 allows MEF2 transcription factor to be active in controlling oxidative fiber determination [56]. Accordingly, we found that HDAC5 transcript was markedly increased in response to HU, in parallel with the decrease in MEF2, in Sol muscle undergoing phenotype transition. While HDAC5 was decreased in IGF-HU mice toward the values found in Sol muscle of WT mice, no restoration was observed in MEF2. This result supports the view that still unclear mechanisms do not allow IGF-1 to exert a full protective action in phenotype preservation during HU, or rather that a stronger modification of HDAC5 activity is required to modify MEF2 expression, as demonstrated in other conditions [57]. Since it has been demonstrated that HDAC5 signaling can be suppressed by the activation of diacylglycerol/PKC and regulated by calcineurin in muscle [58]–[60], we hypothesize that IGF-1-induced repression of HDAC5 may account for the gCl restoration in IGF-HU animals and may represent a first step in the maintenance of the slow phenotype. Further studies are warranted to better evaluate the role of histone deacetylases in disuse-induced muscle impairment and to validate their relevance as pharmacological target. In particular, the determination of protein level of the two IGF-1 sensitive genes MuRF1 and HDAC5 would deserve a focused investigation, also if it has been found that the MuRF1 mRNA modification during atrophy is often accompanied by corresponding changes in protein levels [61]–[63].

Despite the plentiful literature showing the clear role of IGF-1 in muscle hypertrophy and regeneration [17], [64], [65], we found that IGF-1 does not protect all the parameters altered by muscle disuse in the HU model. Various hypotheses can be formulated to explain this apparent discrepancy. For instance, it is possible that, although the number of fast fibers was increased in the HU Sol muscle, IGF-1 was not sufficiently over-expressed in Sol muscle to induce marked effects. Another possibility is that the IGF-1 isoform overexpressed in our mice can be less effective to ameliorate HU condition, compared to other isoforms. We cannot exclude that another IGF-1 isoform specifically involved in load- and stretch-induced adaptation of skeletal muscles, different from those expressed in the liver and in the resting muscle [66], [35], may prove more useful in disuse conditions. Other possibilities include the elevation of the IGF-1-binding proteins, or a reduced expression of the IGF-1 receptor or downstream signaling proteins, possibly due to reduced synthesis or increased protein catabolism during HU, as observed for IRS-1. Such a mechanism may indeed contribute to the limited IGF-1 effectiveness in HU condition. Nevertheless, we also show here that IGF-1 can exert some beneficial effects in HU muscles, at both molecular and functional levels, suggesting a residual activity of IRS-1 or the activation of alternative signaling pathways.

Some studies have underlined the potential of synergistic therapies to increase IGF-1 efficacy as an initiator of protective processes. For instance, GH administration together with resistance exercise attenuates skeletal muscle atrophy in hypophysectomized HU rats [67]. We previously demonstrated that the growth hormone (GH), chronically administrated, allows the improvement of muscles function altered by aging and may also contribute to the partial restoration of excitation-contraction coupling mechanism and fiber diameter in the fast-twitch muscle [68]. Most of the GH effects on muscle involve the local production of IGF-1 [69], which is known to stimulate muscle protein synthesis and to inhibit the catabolic process mediated by atrogenes [38], [70], [71]. It has also been shown that electromechanical stimulation can help to maintain appropriate function of the IGF-1 pathway and muscle mass [72]. At this regard it should be reported that specific exercise training is frequently proposed to the astronauts at the aim to ameliorate muscle dysfunction [2].

In conclusion, mIGF-1 overexpression in 14-days HU mice induces early molecular effects in contrasting muscle atrophy and/or phenotype transition in Sol muscle. Many of the functional parameters, including the electrical and contractile properties, related to phenotype and that are strongly altered in disuse condition, were significantly ameliorated through the paracrine action of the growth factor. Importantly, there is compelling evidence in the literature [67] for the use of combined therapies to fully prevent disuse-induced muscle impairment, and IGF-1 merits further consideration in this context. Finally, we would like to underline the central role that MuRF1 and HDAC5 seem to play in the positive IGF-1 effects which suggest the possibility to develop pharmacological agent acting directly at this level, and that can be more useful to overcome the IGF-1 resistance.
